# Metal Ions Activate the Human Taste Receptor TAS2R7

**DOI:** 10.1093/chemse/bjz024

**Published:** 2019-04-23

**Authors:** Yi Wang, Amanda L Zajac, Weiwei Lei, Carol M Christensen, Robert F Margolskee, Cédric Bouysset, Jérôme Golebiowski, Huabin Zhao, Sébastien Fiorucci, Peihua Jiang

**Affiliations:** 1 Monell Chemical Senses Center, Philadelphia, PA; 2 Department of Ecology and Hubei Key Laboratory of Cell Homeostasis, College of Life Sciences, Wuhan University, Wuhan, China; 3 Université Côte d’Azur, CNRS, Institut de Chimie de Nice UMR7272, Nice, France; 4 Department of Brain and Cognitive Sciences, DGIST, Daegu, Republic of Korea

**Keywords:** TAS2R7, metal ions, bitter taste, metallic taste

## Abstract

Divalent and trivalent salts exhibit a complex taste profile. They are perceived as being astringent/drying, sour, bitter, and metallic. We hypothesized that human bitter-taste receptors may mediate some taste attributes of these salts. Using a cell-based functional assay, we found that TAS2R7 responds to a broad range of divalent and trivalent salts, including zinc, calcium, magnesium, copper, manganese, and aluminum, but not to potassium, suggesting TAS2R7 may act as a metal cation receptor mediating bitterness of divalent and trivalent salts. Molecular modeling and mutagenesis analysis identified 2 residues, H94^3.37^ and E264^7.32^, in TAS2R7 that appear to be responsible for the interaction of TAS2R7 with metallic ions. Taste receptors are found in both oral and extraoral tissues. The responsiveness of TAS2R7 to various mineral salts suggests it may act as a broad sensor, similar to the calcium-sensing receptor, for biologically relevant metal cations in both oral and extraoral tissues.

## Introduction

Divalent salts evoke a complex taste profile, described as metallic, bitter, and astringent (Lim and Lawless 2005). Despite recent progress in the identification of the taste receptor repertoire for sweet and bitter compounds, the molecular mechanisms underlying the complex sensory attributes of divalent salts are largely unknown (Bachmanov and Beauchamp 2007). Using rodent models, Riera et al. (2009) showed that sensory attributes of complex-tasting divalent salts are mediated at least partially by transient receptor potential cation channel subfamily M member 5 (Trpm5) and transient receptor potential vanilloid-1 (Trpv1) channels. Direct activation of Trpv1 by divalent ions may explain the astringency sensation of divalent ions (Riera et al. 2009). Trpm5 is a shared signaling element for sweet, umami, and bitter-taste transduction (Pérez et al. 2002; Zhang et al. 2003). The involvement of Trpm5 for the taste of divalent salts indicates it may be mediated in part by transduction mechanisms similar to that for sweet, bitter, and umami tastes. Interestingly, the sweet and umami receptor subunit T1R3 is reported to be involved in the taste of calcium and magnesium (Tordoff et al. 2008). However, calcium- and magnesium-containing salts are primarily perceived as bitter tasting (Lim and Lawless 2005; Yang and Lawless 2005). Yet how bitterness of these metallic ions is detected is unclear.

Bitter taste is mediated by type 2 taste receptors (TAS2Rs) that are expressed in a subset of taste bud cells (Chandrashekar et al. 2000; Matsunami et al. 2000). TAS2Rs are G protein-coupled receptors (GPCRs) within the rhodopsin family (Chandrashekar et al. 2000; Matsunami et al. 2000). Humans possess 25 functional TAS2Rs. However, the numbers of TAS2R genes vary greatly among mammalian species, ranging from 0 to 54 in amphibian, presumably correlating with the specific ecological niche of a species (Go et al. 2005; Liman 2006; Shi and Zhang 2006; Jiang et al. 2012; Feng et al. 2014; Wang and Zhao 2015; Jiao et al. 2018). Most human TAS2Rs have been deorphanized, and their receptive ranges are heterogeneous (Meyerhof et al. 2010). Some receptors such as TAS2R14 and TAS2R10 are broadly tuned, responding to a wide range of structurally diverse bitter compounds, whereas some others such as TAS2R38 and TAS2R16 are more specialized, responding to relatively few compounds with specific chemical motifs (Kim et al. 2003; Bufe et al. 2005; Meyerhof et al. 2010). This combinatorial TAS2R coding scheme may explain why a relatively limited number of receptors can detect a broad range of structurally diverse bitter compounds.

Given the bitter-taste attribute of multiple divalent salts, we hypothesized that divalent salts may activate one or more TAS2Rs, therefore producing a bitter sensation, contributing to the complex taste attributes of metal ions. To test this hypothesis, we examined which bitter receptor(s) are responsive to divalent salts and found that TAS2R7 responded to all divalent salts tested. In addition, TAS2R7 responded to trivalent salts such as aluminum sulfate. In contrast, potassium chloride, a monovalent salt, does not activate TAS2R7, indicating its specificity. Further structural and functional analyses and molecular modeling revealed H94 and E264 of TAS2R7 as 2 key residues for the receptor’s interaction with metallic ions.

## Materials and Methods

### Preparation of human TAS2R constructs and site-directed mutants

The coding sequences of human TAS2Rs were amplified from human genomic DNA, then subcloned into pcDNA3.1(+) vector, with the herpes simplex virus glycoprotein D epitope (HSV) at the C-terminal and a signal peptide consisting of the first 45 amino acid residues of the rat somatostatin receptor 3 at the N-terminal, essentially as described previously (Bufe et al. 2002). Point mutations in human TAS2R7 (NCBI Reference Sequence: NP_076408.1) were constructed by site-directed mutagenesis. All the constructs were confirmed by Sanger sequencing.

### Chemicals

All tested compounds were purchased from Sigma–Aldrich, with the exception of diphenidol hydrochloride (Reagent World) and L-praziquantel (manufactured by Shaoxing Pharmaceutical Co. Ltd.). All the metal ions were dissolved in the assay solution (130 mM NaCl, 5 mM KCl, 2 mM CaCl_2_, and 10 mM glucose; pH 7.4) unless specified otherwise, and the bitter compounds (diphenidol, quinine, and chlorphenamine) were dissolved first in dimethyl sulfoxide (DMSO) as stock solution and then diluted with the assay solution; the final DMSO concentration was below 0.5%, with the exception of cromolyn, which is dissolved in the assay solution directly. For our initial screening (Table 1), Hanks’ balanced salt solution (HBSS, ThermoFisher, catalog no. 14025134) supplemented with 10 mM hydroxyethyl piperazineethanesulfonic acid (HEPES) were used as the assay buffer. Because HEPES and other buffering agents partially precipitated certain metal ions, the assay solution without buffering agents as described earlier was used for further characterization of TAS2R7.

**Table 1.** Responses of all 25 human TAS2Rs to metallic ions

TAS2R																										
Substance	(mM)	1	3	4	5	7	8	9	10	13	14	16	19	20	30	31	38	39	40	41	42	43	45	46	50	60
ZnSO_4_	20	−	−	−	−	**+**	−	−	−	−	−	−	−	−	−	−	−	−	−	−	−	−	−	−	−	−
CuSO_4_	20	−	−	−	−	**+**	−	−	−	−	−	−	−	−	−	−	−	−	−	−	−	−	−	−	−	−
MgCl_2_	20	−	−	−	−	**+**	−	−	−	−	−	−	−	−	−	−	−	−	−	−	−	−	−	−	−	−

### Functional assays of human TAS2Rs

Human embryonic kidney 293 (PEAKrapid, ATCC # CRL-2828) cells were cultured in Opti-MEM medium with 4% fetal bovine serum. One day before transfection, cells were seeded on a 96-well plate at a density of 25 000 per well. Cells were then transiently transfected with a TAS2R construct (0.1 µg/well) along with a G protein Gα16-gust44 (0.1 µg/well) construct by Lipofectamine 2000 (0.5 µl/well). For controls, only Gα16-gust44 was used (mock transfection). Twenty-four hours after transfection, cells were washed with HBSS including 10 mM HEPES and loaded with Fluo-4 in the dark for 1 h. After incubation, cells were washed 2 times with HBSS (including 10 mM HEPES), incubated in the dark for another 30 min, and then washed with assay solution once more before running the assay using a FlexStation III reader. Relative fluorescence units (excitation at 494 nm, emission at 516 nm, and auto cutoff at 515 nm) were read every 2 s for 2 min. Calcium mobilization traces were recorded.

### Immunostaining

Cells were seeded onto poly-lysine coated coverslips in 24-well plates and transfected with a wild-type or mutant TAS2R7 receptor construct (0.25 µg/well), along with Gα16-gust44 (0.25 µg/well) by Lipofectamine (2.5 µL/well). Twenty-four hour post-transfection, cells were fixed with 4% paraformaldehyde in phosphate-buffered saline (PBS) for 30 min. Cells were then washed with 3 exchanges of PBS and incubated with blocking buffer (2% donkey serum, 0.3% Triton x-100, in SuperBlock [PBS] buffer [ThermoFisher, catalog no. 37515]) for 1 h at room temperature. An anti-HSV antibody (Millipore, catalog no. MAC123, 1:1000) was applied overnight. An Alexa Fluor 488-labled Donkey anti-mouse secondary antibody (Abcam, catalog no. ab150105, 1:1000) were used for fluorescence visualization.

### Data analysis

Calcium mobilization traces were raw data obtained from single wells. Changes in fluorescence (Δ*F*) were calculated as the peak fluorescence minus baseline fluorescence (Lei et al. 2015). The calcium mobilization was quantified as the percentage of change (Δ*F*) relative to baseline (*F*). Each data point for bar graphs and dose-dependent responses was averaged from triplicates (mean ± SD). Calcium mobilization traces and bar graphs along with dose-dependent plots were all generated by GraphPad Prism 7. Analysis of variance with Dunnett’s multiple comparisons test was used for statistical analysis. *P < 0.05.

### Molecular modeling of TAS2R7

The 3D structure of the human TAS2R7 was obtained by comparative modeling using Modeller 9.19 (Sali and Blundell 1993) based on the crystal structure of the 5-HT_2C_ serotonin receptor, PDB identifier 6BQG (Peng et al. 2018) (Supplementary Figure S1). The best homology model according to the DOPE score has been energy minimized using AMBER (Case et al. 2005) and the AMBER ff14SB force field (Maier et al. 2015) parameter prior to structural validation with PROCHECK. Electrostatic potential was calculated with the APBS program (Baker et al. 2001). To obtain accurate electrostatic properties, we used the 2-step focusing technique and a grid spacing lower than 0.5 Å in each space dimension. The molecular surface was generated using a water probe with a radius of 1.4 Å. The dielectric constant of the protein and the solvent was fixed to 2 and 80, respectively. The protonation states of titratable residues were predicted at pH 6.5 through the H++ server (Gordon et al. 2005). Cromolyn was docked within the TAS2R7 binding cavity using Autodock Vina (Trott and Olson 2010). The Zn^2+^ cation was manually docked into the TAS2R7 model. The cation-receptor complex was energy minimized with the AMBER software using 500 steps of the steepest descent optimization followed by 1000 steps of conjugate gradient optimization with positional restraints of 50 kcal/mol/Å on backbone heavy atoms.

## Results

### Identification of TAS2Rs for metal ions

**Figure 1.**
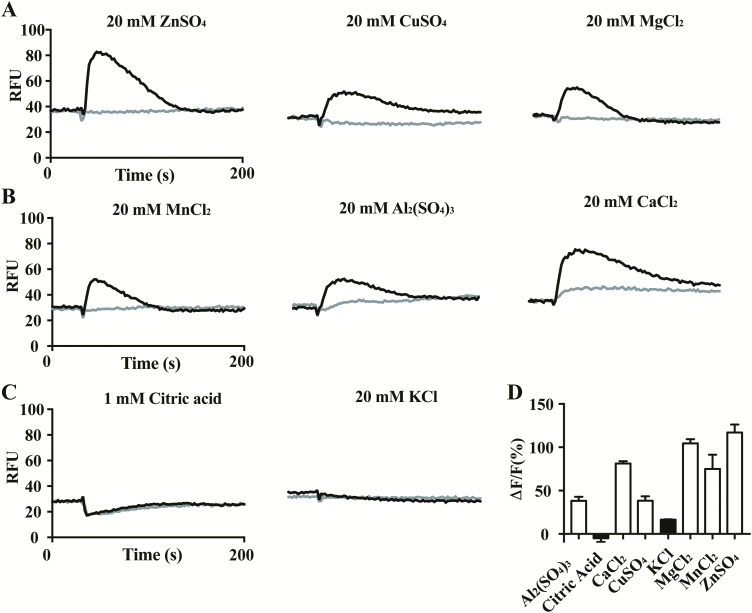
Metal ions activate TAS2R7. (**A–C**) HEK293 cells transfected with human TAS2R7 with Gα16-gust44 were assayed for their responses to metal ions and citric acid. Black traces, representative calcium mobilization traces of TAS2R7 to compounds; gray traces, mock-transfected cells used as control. RFU, relative fluorescence unit. (**D**) Quantitative analysis of responses of TAS2R7 to metallic ions and citric acid. Data are percentage change (mean ± SD) in fluorescence (peak RFU − baseline RFU, denoted Δ*F*) from baseline fluorescence (denoted F) averaged from triplicates. Experiments were replicated 3 times.

To determine whether a TAS2R responds to metal ions, we expressed all 25 human bitter receptors individually in HEK293 cells (PEAKrapid) by transient transfection of a TAS2R along with a coupling chimeric G protein, Gα16-gust44. All TAS2Rs were cloned from human genomic DNA. Activation of human TAS2Rs was monitored by the calcium mobilization assay (Lei et al. 2015). We tested these receptors individually for their responses toward metal ions: ZnSO_4_ (20 mM), CuSO_4_ (20 mM), and MgCl_2_ (20 mM) (Table 1, Figure 1A). No receptors showed responsiveness to these metal ions, with the exception of TAS2R7, which consistently showed robust responses toward all 3 divalent salts. To determine the breadth of tuning of TAS2R7 toward metal ions, we also tested MnCl_2_ (20 mM), Al_2_(SO_4_)_3_ (20 mM), and CaCl_2_ (20 mM) (Figure 1B). All divalent and trivalent ions activated the receptor, albeit with variable degrees of efficacy (Figure 1A,B). ZnSO_4_ solution is acidic (pH ~5) at the concentration we tested, as is Al_2_(SO_4_)_3_ solution (pH ~3). To determine whether pH affected the activity of TAS2R7, we tested the responsiveness to TAS2R7 to 1 mM citric acid (pH ~3) (Figure 1C). No specific response was detected. Therefore, the responses of TAS2R7 toward metal ions were specific. In contrast to divalent and trivalent cations, the monovalent salt KCl did not activate the receptor, suggesting that TAS2R7 is specifically tuned to divalent and trivalent salts (Figure 1C,D). To determine whether anions might affect the potency and efficacy of cations, we compared the responses of TAS2R7 toward ZnSO_4_ and ZnCl_2_. No obvious differences were found between 2 types of anions (EC_50_ of ZnSO_4_: 3.21 mM, ZnCl_2_: 3.42 mM) (Supplementary Figure S2). To our knowledge, aside from calcium-sensing receptor (CaSR), TAS2R7 is the only GPCR that can be activated by multiple metal ions (Brown et al. 1993; McGehee et al. 1997; Saidak et al. 2009).

### TAS2R7 responds to metal ions in a dose-dependent manner

**Figure 2.**
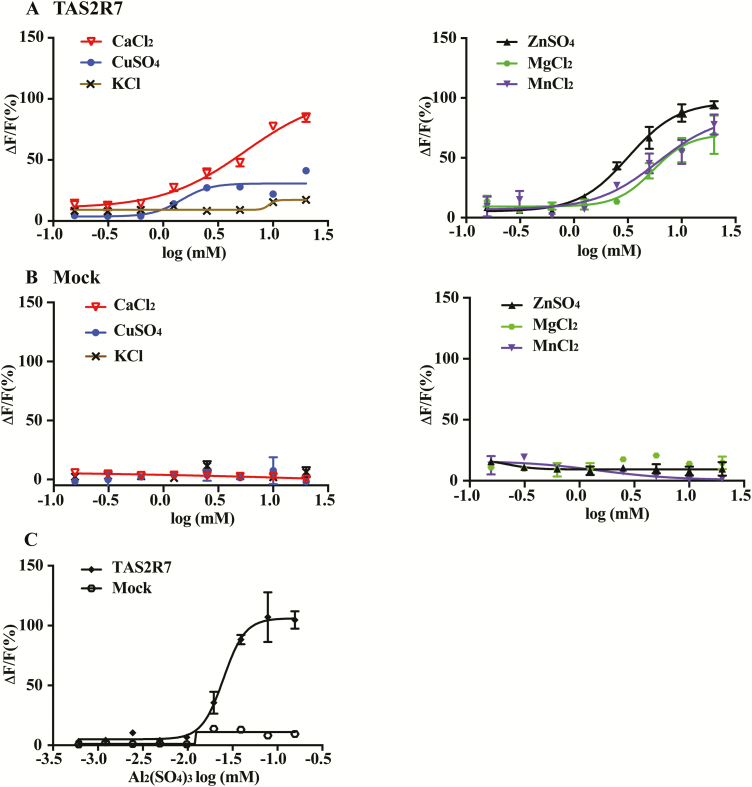
TAS2R7 responds to metal ions dose dependently. HEK293 cells transiently transfected with human TAS2R7 with Gα16-gust44 showed dose-dependent responses to metal ions: CaCl_2_, CuSO_4_, ZnSO_4_, MgCl_2_, MnCl_2_, and Al_2_(SO_4_)_3_ (**A**, **C**). KCl does not activate TAS2R7 at any concentrations tested (A, left panel). Mock-transfected cells (Gα16-gust44 only, Mock) were used as controls for cell transfected with TAS2R7 in response to metal ions (**B**, **C**). GraphPad Prism 7 was used to fit the curve (sigmoidal). Experiments were replicated 3 times.

To determine the sensitivity of TAS2R7 toward metal ions, we generated concentration–response functions (Figure 2). TAS2R7 responded to all metal ions we tested in a dose-dependent manner (Figure 2A), whereas mock-transfected cells showed no responses to metal ions at any concentration we tested (Figure 2B). Nevertheless, the efficacy differs among different cations. The receptor appears to be most sensitive toward aluminum sulfate (EC_50_, 39 ± 15 µM), followed by CuSO_4_ (EC_50_, 1.04 ± 0.36 mM), ZnSO_4_ (EC_50_, 33.36 ± 0.14 mM), MgCl_2_ (EC_50_, 6.07 ± 1.07 mM), CaCl_2_ (EC_50_, 5.27 ± 0.50 mM), and MnCl_2_ (EC_50_, 6.59 ± 1.73 mM). Mock-transfected cells showed no responses to any concentration of Al_2_(SO_4_)_3_ tested. As expected, the receptor was also not responsive to any concentration of KCl. Thus, TAS2R7 interacts differently with different ions.

**Figure 3.**
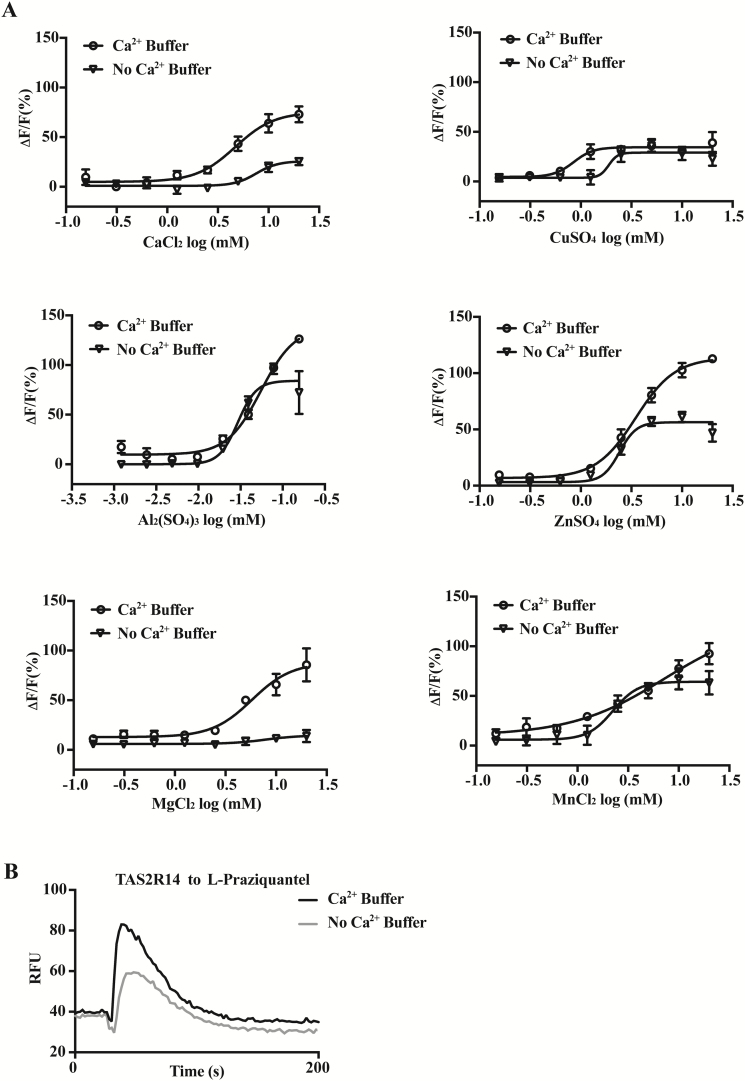
Responses of TAS2R7 to metal ions in the absence and presence of calcium in the assay solution. (**A**) Responses of HEK293 cells transiently transfected with human TAS2R7 with Gα16-gust44 to 6 metal ions in the presence and absence of calcium in the assay solution, including CaCl_2_, CuSO_4_, ZnSO_4_, MgCl_2_, MnCl_2_, and Al_2_(SO_4_)_3_ respectively. GraphPad Prism 7 was used to draw the dose-dependent curves. (**B**) TAS2R14 was expressed along with Gα16-gust44 in the HEK293 cells, and the responses to 0.5 mM L-praziquantel were assayed with the presence and absence of calcium. Black traces, calcium mobilization with the presence of calcium; gray traces, with the absence of calcium. Experiments were replicated twice.

Our assay solution contains 2 mM calcium ion, which supports optimal assay condition for the calcium mobilization assay, yet TAS2R7 responds to calcium. Therefore, to determine whether the presence of calcium affects the responses of TAS2R7 to metal ions, we performed calcium mobilization assays using assay solution containing no calcium (130 mM NaCl, 5 mM KCl, and 10 mM glucose; pH 7.4). All the tested compounds were dissolved in the same assay solution. As expected, TAS2R7 showed robust responses to all 6 metal ions tested under this condition (Figure 3A). Concentration-dependent curves were similar in the presence and absence of calcium in the assay solution. The EC_50_ of 6 metal ions in the absence of calcium is as follows: CaCl_2_, 4.70 mM; CuSO_4_, 0.85 mM; ZnSO_4_, 3.49 mM; MgCl_2_, 5.78 mM; MnCl_2_, 7.19 mM; Al_2_(SO_4_)_3_, 55 µM, respectively, similar to the EC_50_s in the presence of calcium (CaCl_2_, 7.56 mM; CuSO_4_, 1.89 mM; ZnSO_4_, 2.41 mM; MgCl_2_, 7.84 mM; MnCl_2_, 2.24 mM; Al_2_(SO_4_)_3_, 29 µM). However, the maximal responses to all metal ions were smaller in the absence than in the presence of calcium, especially toward MgCl_2_. This appears to be a general phenomenon for this type of assay, as shown by reduced response amplitude for other GPCRs as well (e.g., TAS2R14 to L-praziquantel, Figure 3B). All the dose-dependent curves were replicated at least twice.

### TAS2R7 is a narrowly tuned receptor

**Figure 4.**
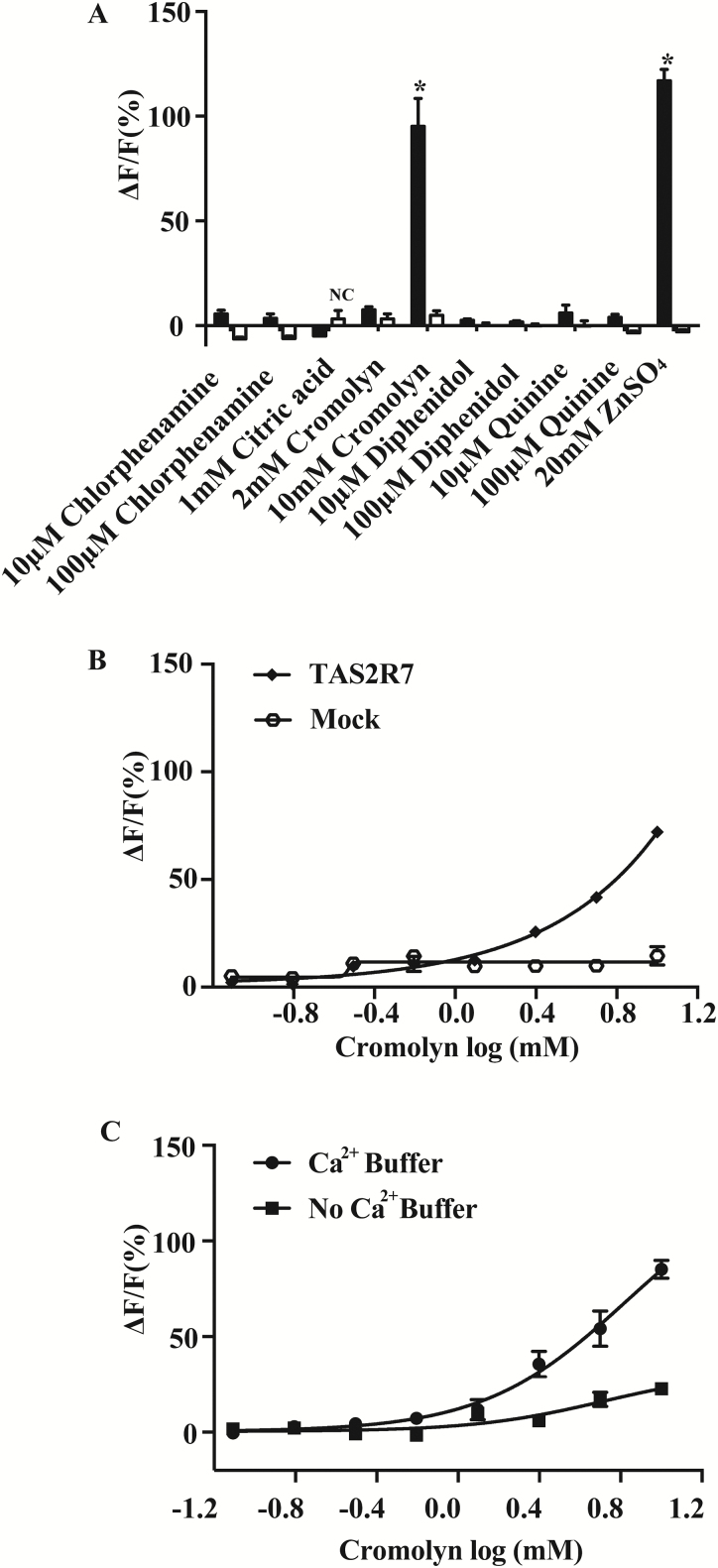
TAS2R7 is a narrowly tuned receptor. HEK293 cells were transiently transfected with human TAS2R7, coupled with Gα16-gust44, and their responses assayed to previously reported bitter ligands. Two-tailed *t*-tests were used to determine whether there is a significant difference between the TAS2R7-transfected cells and mock-transfected (Gα16-gust44 only) cells. (**A**) Responses to ZnSO_4_ and citric acid were chosen as positive control and negative control (NC), respectively. Bitter compounds that stimulate significant responses are indicated with an asterisk (*) (*P* < 0.05). (**B**) Cromolyn activates TAS2R7 in a dose-dependent manner. Experiments were replicated 3 times. (**C**) Dose-dependent curves of TAS2R7 toward cromolyn with the presence and absence of calcium. Experiments were replicated twice.

TAS2R7 has been reported to respond to certain bitter compounds, including diphenidol, quinine, cromolyn, and chlorphenamine (Meyerhof et al. 2010). To further determine the tuning properties of TAS2R7, we examined its responsiveness to bitter compounds that were previously shown to activate the receptor (Figure 4A) (Meyerhof et al. 2010). At the concentrations reported previously, none of the compounds we tested (diphenidol, quinine, cromolyn, and chlorphenamine) triggered detectable responses in cells transiently transfected with TAS2R7 in our hands (Meyerhof et al. 2010). However, cromolyn at a higher dose (10 mM) did elicit a robust response in cells specifically transfected with TAS2R7 but not in mock-transfected cells. We further confirmed the requirement of high doses of cromolyn to activate the receptor by dose-response analysis (EC_50_, 5.9 mM) (Figure 4B). For other compounds, even higher doses produced no responses (Figure 4A). Thus, our data indicate that TAS2R7 selectively responds to metal ions and cromolyn. We also performed cell-based assay with the presence and absence of calcium for cromolyn (Figure 4C). As expected, the maximal response is smaller using the assay solution containing no calcium than the assay solution containing calcium, while the EC_50_s are comparable (with calcium: 6.67 mM; without calcium: 5.22 mM). Therefore, we used assay solution containing calcium for our further analysis of the receptor to have a better readout.

### Molecular modeling and site-directed mutagenesis identify 2 residues of TAS2R7 critical for the recognition of metal ions

**Figure 5.**
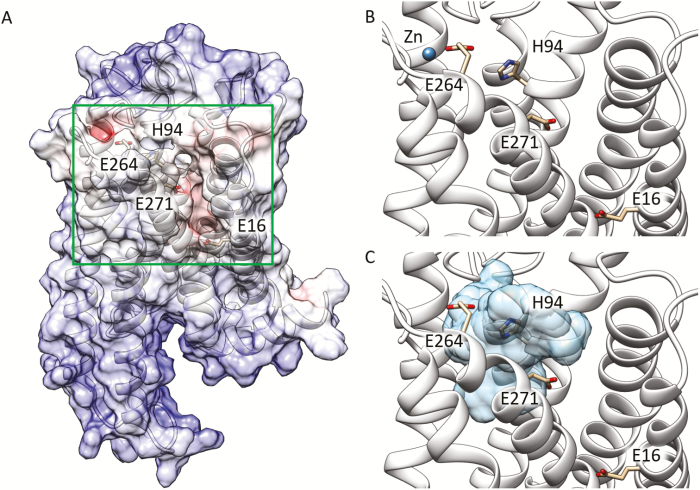
Molecular model of TAS2R7’s binding pocket with docked ligands. (**A**) Electrostatic potential (±10 kbT/e) mapped onto the molecular surface of the protein. Red and blue colors indicate negatively and positively charged regions, respectively. The most attractive cavity for cation binding is delimited by the green box. (**B**) Minimized structure of TAS2R7 interacting with Zn^2+^. (**C**) Binding cavity of TAS2R7 (in light blue) explored by cromolyn in the docking simulations.

To predict how TAS2R7 interacts with metal ions, a homology model of TAS2R7 was built based on the crystal structure of the 5-HT_2C_ serotonin receptor (Peng et al. 2018). We first automatically docked cromolyn into the GPCR binding cavity formed by helices 2, 3, 5, 6, and 7 because metal ions are too small for initial docking simulations (Figure 5C). The results of docking simulations identified a pocket similar to that defined by Liu et al. (2018). All amino acids involved in contact with the ligand are part of the typical TAS2R binding pocket (Supplementary Figure S3). The electrostatic potential computed on the TAS2R7 model shows a negatively charged region (Figure 5A) suitable for attracting cations. Accordingly, negatively charged or polar residues in this area, E16^1.42^, H94^3.37^, E264^7.32^, and E271^7.39^ (the superscripts refer to the Ballesteros–Weinstein notation; Ballesteros and Weinstein 1995), are considered to interact with metal ions through strong electrostatic interactions (Figure 5B).

**Figure 6.**
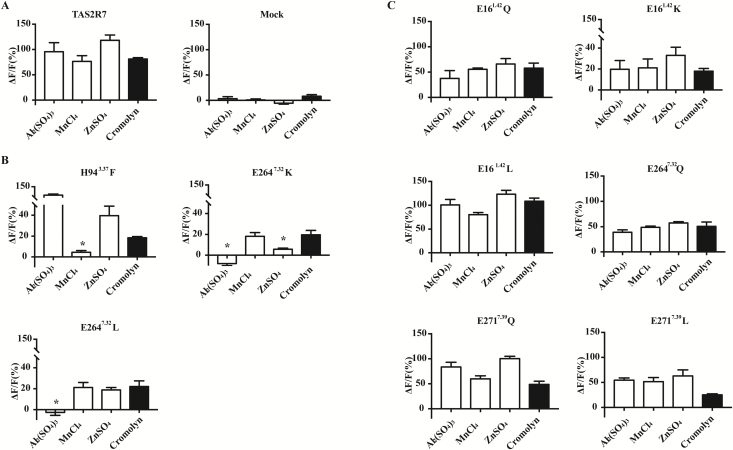
Mutagenesis analysis of the predicted binding pocket for metal ions. Wild-type (**A**) and mutant receptors (**B**, **C**) were expressed along with Ga16-gust44 in HEK293 cells, and their responses to metal ions and cromolyn were examined. (B) Mutant receptors showing selectively reduced responses to certain metal ions. (C) Mutant receptors showing no specific reduction in responses to metal ions. Dunnett’s multiple comparisons test was performed to determine when the responses to metallic ions of mutants were significantly decreasing from that of cromolyn, indicated with an asterisk (*) (*P* < 0.05). Experiments were replicated 3 times.

To assess the importance of these residues, we performed site-directed mutagenesis. We mutated the negatively charged residues E16^1.42^, E264^7.32^, and E271^7.39^ to Q (glutamine), K (lysine), or L (leucine). The facing H94^3.37^ was mutated to F (phenylalanine). HEK293 cells that expressed mutant receptors along with Gα16-gust44 were examined for their responses to metal ions (20 mM for all except 0.16 mM Al_2_(SO_4_)_3_) and cromolyn (10 mM, as a positive control) to assess receptor’s function. To determine the expression level of each receptor, we stained the wild-type or mutant receptor-transfected cells using an anti-HSV antibody since all the receptors are tagged with HSV at c-terminal. There was no obvious difference in the intensity of the staining among mutants and wild-type receptors (Supplementary Figure S4). Compared with the wild-type receptor (Figure 6A), 2 classes of mutants were noted: those showing significantly diminished responses to only a subset of metal ions (Figure 6B), and those showing either normal or reduced responses to both metal ions and cromolyn (Figure 6C).

For example, H94^3.37^F showed diminished responses specifically MnCl_2_ (Figure 6B). In contrast, E264^7.32^K showed specific loss of responses to ZnSO_4_ and Al_2_(SO_4_)_3_, and E264^7.32^L responded to ZnSO_4_ but not to Al_2_(SO_4_)_3_. With both E264^7.32^K and E264^7.32^L, the overall responses of mutant receptors to metal ions and cromolyn were reduced. Similarly, substitution of glutamate with glutamine (E264^7.32^Q) led to a mutant receptor showing reduced responses to metal ions and cromolyn but did not specifically affect the receptor response to a particular metal ion. Substitution of glutamate at E16^1.42^ with other residues showed no specific effects on the activity of metallic ions. However, with the exception of E16^1.42^L, all other mutations led to relatively smaller responses to both metal ions and cromolyn compared with wild-type TAS2R7. Substitution of E271^7.39^ with either glutamine or leucine led to a mutant receptor showing slightly reduced responses to all metal ions and cromolyn in vitro. Together, our mutagenesis data suggest the involvement of H94^3.37^ and E264^7.32^ in interacting with metal ions.

## Discussion

### TAS2R7 as a metal ion detector

By systematically assaying all the human bitter receptors for their responsiveness to metal ions, we found that TAS2R7 acts as a receptor for divalent and trivalent cations. To our knowledge, only CaSR and GPR39 have been previously shown to be metal-sensing receptors (Brown et al. 1993; McGehee et al. 1997; Holst et al. 2007; Saidak et al. 2009). Identification of TAS2R7 as a metal-ion-sensing receptor broadens our understanding how metal ions are sensed.

TAS2Rs evolved to detect bitter substances (which are potentially harmful or toxic) in diets. Activation of these receptors would then induce aversive behavior as a defense mechanism (Bachmanov and Beauchamp 2007). Most natural compounds that taste bitter are plant derived. Some plants are known to be rich in minerals. Vegetable bitterness is shown to be related to calcium content (Tordoff and Sandell 2009). Thus, activation of TAS2R7 may contribute to bitterness associated with calcium-rich (or mineral-rich) vegetables. Future work is warranted to determine if blocking TAS2R7 (e.g., inhibitors of TAS2R7) can reduce bitterness or metallic taste of metal ions or mineral-rich foods.

Taste disturbance is a widely reported side effect for cancer patients who receive chemotherapy or radiotherapy (Comeau et al. 2001). Often, they complain about bitter taste or metallic taste (Comeau et al. 2001). It is conceivable that such treatments may alter bitter receptor gene expression, such as upregulation of TAS2R7 that is normally expressed at a low level. Metal ions in the blood may activate the receptor, leading to bitter/metallic taste perception in pathological conditions. Blocking TAS2R7 activity may provide a therapeutic strategy for alleviating chemotherapy- or radiotherapy-induced taste disturbance.

### Interaction of metal ions and TAS2R7

Our structure–function analysis of TAS2R7 showed differential requirements of H94 in helix 3 and E264 in helix 7 for their interaction with different metal ions. Substitution of the histidine residue at position 94 (H^3.37^) with phenylalanine diminished responsiveness of the receptor toward MnCl_2_ more than toward Al_2_(SO_4_)_3_, ZnSO_4_, and cromolyn in vitro. Conversely, substitution of the negative-charged glutamate residue at position 264 (E^7.32^) with positive-charged lysine rendered the receptor insensitive to Al_2_(SO_4_)_3_ and ZnSO_4_ but still responsive to MnCl_2_. Similarly, substitution with the neutral but slightly bulkier leucine residue also rendered the receptor insensitive to Al_2_(SO_4_)_3_. Altogether, our demonstration of the contribution of H94 and E264 to a binding pocket for metal ions is supported by both mutagenesis analysis and molecular modeling. In addition, we showed that these ions interact distinctively with residues lining this binding pocket. Especially, the presence or absence of calcium in the assay solution appears to influence the responses of TAS2R7 distinctly for different metal ions. We do not know the reason but speculate that calcium may work cooperatively with certain ions (e.g., ZnSO_4_, MgCl_2_) than with others (e.g., CuSO_4_). Future detailed structure–function analysis of interactions of the receptor and metal ions will provide further insights into how metal ions activate the receptor.

### Potential extraoral function of TAS2R7

Recently, TAS2Rs have been shown to be expressed not only in the oral cavity but also in many other tissues in the body (Behrens and Meyerhof 2011). However, the endogenous cognate ligands for these extraoral receptors are largely unknown. Compared with other TAS2Rs, TAS2R7 is reported to be weakly expressed in taste bud cells (Behrens et al. 2007). Using immunostaining and RT-PCR, it has been shown that TAS2R7 is also expressed in pancreatic islet cells (Chen et al., 2007).

Zinc is known to be an important regulator of islet function. Pancreatic β cells contain high concentrations of zinc in the secretory granules (Wijesekara et al. 2009). Upon excitation of β cells, Zn^2+^ is coreleased at high concentrations with insulin into the extracellular space of the islet. Given the presumptive expression of TAS2R7 in a subset of islet cells, it is tempting to speculate that the released Zn^2+^ may act on TAS2R7-expressing cells to regulate glucose homeostasis. Indeed, using human genetic approaches, Dotson et al. (2008) showed that a nonsynonymous coding SNP in TAS2R7 is associated with type 2 diabetes mellitus. However, we found no significant difference in the responsiveness of TAS2R7 having isoleucine residue at the position 304 and the receptor carrying M304 toward divalent and trivalent metals (data not shown).

There is compelling evidence supporting that extracellular Al^3+^ at micromolar concentrations activates a GPCR-like signaling pathway in certain cells (Spurney et al. 1999). Aluminum has been shown to be a weak agonist for CaSR (Spurney et al. 1999). Given the efficacious response of TAS2R7 toward Al^3+^, it is possible that TAS2R7 mediates certain biological responses elicited by aluminum ions. Indeed, Al^3+^ administered systemically can reach 50 µM in serum in animal studies and stimulates osteoblast-mediated de novo bone formation in vivo and osteoblast proliferation in vitro (Lau et al. 1991). This is within the sensitivity of TAS2R7 to Al^3+^ (Figure 2).

Another study performed by Velázquez-Fernández et al. (2006) showed that TAS2R7 is upregulated in parathyroid adenoma samples compared with parathyroid hyperplasia samples, suggesting a potential link between TAS2R7 and regulation of calcium homeostasis. However, CaSR acts as a principal regulator of calcium homeostasis.

CaSR is known to respond to a variety of divalent and trivalent ions (18–20). Despite the similarity in the responses to divalent and trivalent ions of CaSR and TAS2R7, differences between these 2 receptors are notable. For example, TAS2R7 responds to zinc ions, and CaSR does not. Thus, in terms of specificity for metal ions, TAS2R7 appears to be more broadly tuned. The physiological role of TAS2R7 in extraoral tissues and the possibility of metal ions as its endogenous ligands warrant future investigation.

## Funding

This work was supported in part by a grant from the Bill and Melinda Gates Foundation (OPP1159241). Calcium assays were performed at the Monell Chemosensory Receptor Signaling Core, which was supported in part by NIH–National Institute on Deafness and Other Communication Disorders Core Grant DC011735 (R.F.M.). Y.W. was partially supported by the China Scholarship Council, and H.Z. was supported by the National Natural Science Foundation of China (31722051). The content is solely the responsibility of the authors and does not necessarily represent the official views of the National Institutes of Health.

## Supplementary Material

bjz024_Suppl_Supplementary_Figures
