# Supplementary material for: Metal Ions Activate the Human Taste Receptor TAS2R7
Source: Chem Senses. 2019 Apr 23;44(5):339–47. doi: 10.1093/chemse/bjz024 (PMC6538953; doi:10.1093/chemse/bjz024)
Supplement: bjz024_Suppl_Supplementary_Figures [file bjz024_suppl_supplementary_figures.docx]

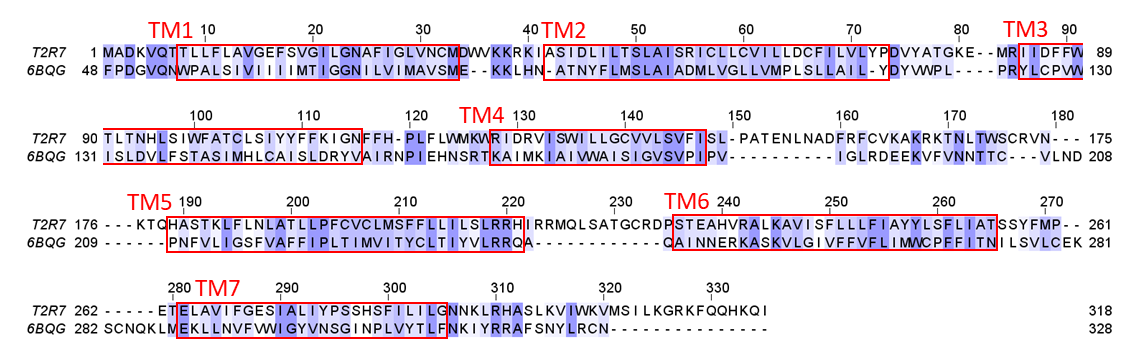


**Figure S1. Alignment of TAS2R7 and 5-HT_2C_ serotonin receptor (PDB 6BQG) sequences**. Transmembrane helices are delimited by red boxes. Conserved residues are shown in dark blue. Aligned residues with a positive Blosum62 score are shown in light blue.


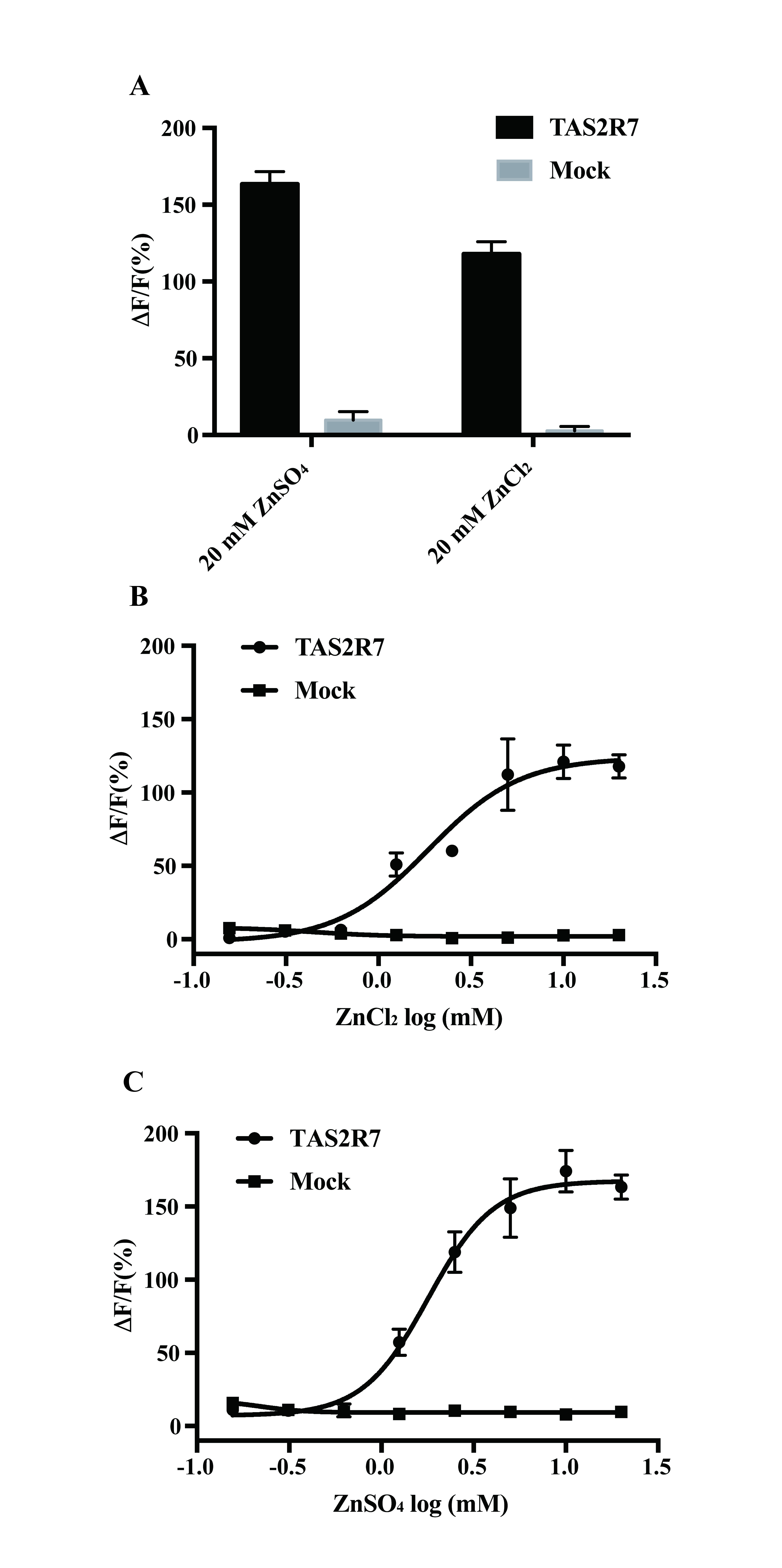


**Figure S2**. **Responses of TAS2R7 toward ZnCl_2_ and ZnSO_4_.**

Responses of HEK293 cells transiently transfected with human TAS2R7 with Gα16-gust44 to ZnSO_4_ and ZnCl_2_, respectively. A) Quantitative analysis of responses of TAS2R7 to 20 Mm ZnSO_4_ and ZnCl_2_. Data are percentage change (mean ± SD) in fluorescence (peak RFU – baseline RFU, denoted ΔF) from baseline fluorescence (denoted F). Experiments were replicated three times. B, C) Dose-dependent curves of TAS2R7 toward ZnSO_4_ and ZnCl_2_, Experiments were replicated twice.


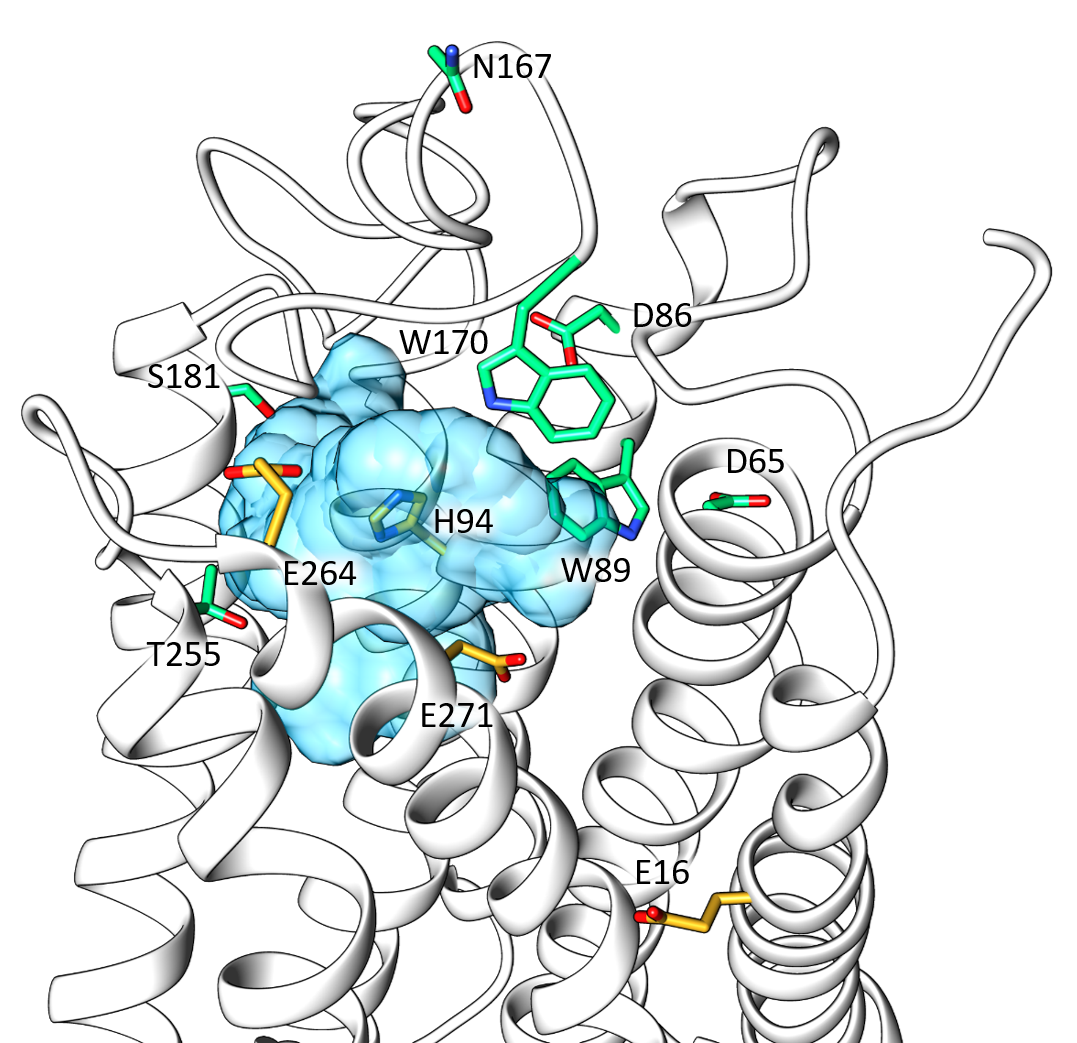


**Figure S3.** Comparison of our TAS2R7 homology model with a previously published model. The binding cavity of TAS2R7 (in blue) was explored with cromolyn during the docking simulations. Residues proposed by Liu *et al.* (21) to be part of the binding pocket are shown in green: D65, D86, W89, N167, W170, S181, T255, E271. Residues affecting metallic interaction suggested by the present study are shown in yellow: E16, H94, E264, E271.

**Figure S4**. **Immunostaining of cells transfected with mutants and wildtype TAS2R7 receptors.** HEK293 cells expressing TAS2R7 or its mutants were immunostained with an anti-HSV antibody. An Alexa Fluor 488-labled Donkey anti-mouse secondary antibody was used for fluorescence visualization (Green). A brightfield image of the same field was shown next to the fluoresent image. Images were taken with the same exposure time and the same setting.
